# Accelerating the search for the missing proteins in the human proteome

**DOI:** 10.1038/ncomms14271

**Published:** 2017-01-24

**Authors:** Mark S. Baker, Seong Beom Ahn, Abidali Mohamedali, Mohammad T. Islam, David Cantor, Peter D. Verhaert, Susan Fanayan, Samridhi Sharma, Edouard C. Nice, Mark Connor, Shoba Ranganathan

**Affiliations:** 1 Department of Biomedical Sciences, Faculty of Medicine & Health Sciences, Macquarie University, New South Wales 2109, Australia; 2 Department of Chemistry & Biomolecular Sciences, Macquarie University, New South Wales 2109, Australia; 3 Department of Biology, Antwerp University, Antwerpen 2020, Belgium; 4 Department of Biochemistry and Molecular Biology, Monash University, Victoria 3800, Australia

## Abstract

The Human Proteome Project (HPP) aims to discover high-stringency data for all proteins encoded by the human genome. Currently, ∼18% of the proteins in the human proteome (the missing proteins) do not have high-stringency evidence (for example, mass spectrometry) confirming their existence, while much additional information is available about many of these missing proteins. Here, we present MissingProteinPedia as a community resource to accelerate the discovery and understanding of these missing proteins.

## Abstract (web summary)

The Human Proteome Project aims to catalogue the ∼20,000 proteins encoded by the human genome. In this review, Baker *et al*. focus on the missing proteins, proteins that lack high stringency proteomic evidence, and launch MissingProteinPedia, a database aimed at accelerating the search for missing proteins.

The Human Proteome Project (HPP) supports defining what it is to be human in molecular terms. It strives to ‘know thyself' by finding high-stringency evidence for the ∼20,000 proteins encoded by the human genome. Here, we focus on what has been termed the human proteome's ‘missing proteins', discuss what renders them currently unobservable using high-stringency proteomic approaches, and outline a road-map that aims to accelerate the HPP. We review milestones and the progress of this global scientific effort to accurately identify and understand the biology of genome-coded human proteins. We focus on what has been achieved to date and we identify some areas where progress may be made. We provide a comprehensive survey of the characteristics of the so-called ‘missing proteins', a term initially coined by Hancock and colleagues defined in Box 1 (refs 1, 2), and we emphasize why they may be difficult to detect using mass spectrometry (MS) and/or validated antibody (Abs) techniques. Our re-analysis of publicly available MS data for the largest family of missing proteins (olfactory receptors), viewed in conjunction with other specific missing protein examples reveals a need for the community to capture as much complementary evidence as possible about missing proteins, in addition to high-stringency MS data. With this aim, we launch MissingProteinPedia ( http://www.missingproteins.org), a community biological database that is complementary to the high-stringency HPP methodologies currently underway. MissingProteinPedia is a low-stringency communal database that will increase our understanding of the spatiotemporal biology of missing proteins, and accelerate their discovery by high-stringency MS.

## Human Proteome Project (HPP) goals and progress

Science is rapidly becoming a global endeavour, with high-quality curation and annotation of data becoming the responsibility of the whole scientific community. Despite the Delphic maxim ‘know thyself' being inscribed on the forecourt of the Temple of Apollo in ancient Greece during the sixth century BC, we still do not have a comprehensive description of what it means to be human in strictly molecular terms (that is, genome + epigenome + transcriptome + proteome + peptidome + metabolome). In 2010, the Human Proteome Organization (HUPO) formally initiated a flagship project called the Human Proteome Project (HPP). This ambitious project contributes to humans knowing themselves by collecting credible, high-stringency MS and other evidence for the ∼20,000 or so proteins coded by human genes. The long-term aims of HPP are twofold. First, it aims to complete the protein ‘parts list' of *Homo sapiens* by identifying and characterizing at least one protein product and as many post-translational modifications, single amino acid polymorphisms and splice variant isoforms as possible for each protein-coding gene. Second, it aims to transform proteomics so it becomes complementary to genomics across clinical, biomedical and life sciences, through technological advances and creation of knowledgebases for the identification, quantitation and characterization of the functionally networked human proteome.

In order to ensure all encoded proteins would be revealed and that all important biology and diseases would be represented, the HPP was amalgamated under two distinct but overlapping streams called the chromosome-centric (C-HPP) and Biology/Disease (B/D-HPP) Human Proteome Projects^3^. These are underpinned by three resource pillars; (i) MS, (ii) Affinity Reagents (for example, Abs), and (iii) a Knowledgebase. In addition to re-analysing and reporting HPP data, a number of complementary groups (PeptideAtlas; http://www.peptideatlas.org, neXtProt; http://www.neXtProt.org, GPMDB; http://www.gpmdb.org and Human Protein Atlas (HPA); http://www.proteinatlas.org) work cooperatively to provide annual HPP updates, present chromosome-by-chromosome tabulations, evolve high-stringency HPP data analysis metrics^4^,^5^, and supply HPP data deposition guidelines for all researchers^6^. Critically, the HPP consortium encourages concurrent raw data deposition through standardized MS portals (for example, ProteomeXchange; shown as a schema in Box 2). The HPP also undertakes critical, annual re-analyses and reporting of the growing MS dataset with accompanying metadata using community-approved, high-stringency metrics.

The desire to build a reproducible, definable, metrics-driven, annotated HPP of the highest quality necessitated the imposition of terms defining the categories of evidence obtained. To enable this, it was communally agreed that the protein-centric knowledge platform neXtProt^7^,^8^ would classify HPP proteins by protein existence (PE), based on partial/complete Edman sequencing, identification by MS, 3D structure (X-ray/NMR), good quality protein–protein interaction data and/or detection of a protein by validated Abs (for example, in the HPA^9^). Metrics, guidelines and/or PE categories have been agreed on and revised through community forums, facilitated by HUPO. Since the HPP was launched in 2010, we have learned many lessons. The importance of ‘speaking the same language' with regard to MS analysis metrics and data submission guidelines has been prominent. Kim *et al*.^10^ and Wilhelm *et al*.^11^ proposed drafts of the human proteome in 2014. These studies challenged the imposition of communal metrics, including previously agreed consensus regarding protein target-decoy false discovery rates (FDRs) and requisite minimum proteotypic peptide length (≥7 amino acids in 2014). The term proteotypic in this context refers to a human peptide sequence of any length found by MS that is uniquely derived from a single known human protein expressed by the genome. The term is often used interchangeably with the commonly used terms, uniquely expressed and unitypic. In the HPP, proteotypic peptides (that is, two proteotypic peptides of suitable length) are employed to identify the expression of a human protein by MS methods. Discussion around the impact of single amino acid variation on application of the term proteotypic are currently underway.

Conclusions from both the human proteome drafts^10^,^11^ were considered contentious^12^,^13^ because they chose to interrogate MS findings using different metrics to those established by the HPP after communal agreement. Because of debate around these publications, large-scale heterogeneous datasets were recognized as raising questions related to assumptions around FDR protocols^12^. Encouragingly, positive, collaborative, communal efforts (for example, revised data deposition guidelines and clear diagrammatic representations of data re-analysis workflows and metrics) are underway and will resolve many of the issues raised. In response, the HPP Knowledgebase pillar proposed more rigorous metrics for substantiating claims of the identification of previously unobserved proteins (that is, PE2-5 proteins; Box 1). It has been proposed that datasets should be culled at 1% protein FDR with additional estimates of peptide and peptide spectral match (PSM) level FDRs and notification of the numbers of proteins, peptides and spectra passing/failing these thresholds. In late 2015, PeptideAtlas proposed increasing the minimum thresholds to two proteotypic peptides of ≥9 amino acids with raw spectra to be made publicly available (downgrading 432 previously validated PE1 proteins)^4^. Some exceptions included predicted proteins that are unable to be cleaved to form at least two tryptic proteotypic peptides of required length^4^. While neXtProt initially retained less stringent criteria thresholds of two proteotypic peptides of ≥7 amino acids or one proteotypic peptide of ≥9 amino acids (that is, with consequent downgrading of 20 PE1 proteins), in February 2016 they aligned with the more stringent PeptideAtlas metrics. These developments were incorporated into both the 2016 HPP metrics and HPP guidelines for data submission that have been recently published^5^,^6^. It should be noted that while the observation of two ≥9 amino acid proteotypic peptides by highly accurate MS dramatically reduces statistical uncertainty, it does not make the putative identification of any protein unequivocal.

## What is known about missing proteins

On behalf of the HPP, neXtProt curates, integrates and computes PE (PE1-5) scores based on experimental information from multiple types of enquiry (see Box 1). In this review, we focus solely on those proteins that are classified as being either PE2 (evidence only at transcript level), PE3 (inferred from homology) or PE4 (proteins inferred to exist). These three PE groups have been collectively and colloquially defined as the HPP ‘missing proteins'^1^,^2^ (Box 1), although a recent study erroneously mentions missing proteins include PE5s^14^, which are highly unlikely to be translated. Definitions for PE1-5 (ref. 4 ) proteins are released by neXtProt before annual HUPO Congresses.

The HPP endorses open, community-wide use of standardized re-analysis pipelines, with attention to the evolving HPP guidelines for researcher data submission^6^ and metrics used for global concatenated communal data re-analyses^4^,^5^. It also encourages confirmation of novel findings with advanced MS methods (for example, selected reaction monitoring (SRM) and data-independent acquisition, including new methods such as SWATH-MS^15^). This process implies that PE2-4 proteins need to be re-classified regularly (that is, upgraded or downgraded) after agreed, metrics-driven, communal re-analysis, preferably with publication of the rationale for their re-assignment. This high-stringency approach is crucial for quality assurance and is favoured over any individual laboratory MS data analysis, that can result in potentially contestable claims that regularly arise for ‘finding' suites (sometimes hundreds) of PE2-4 missing proteins.

It should be stressed that the PE2-4 proteins only represent a list of proteins currently not fulfilling HPP metrics, and that these lists have evolved since the launch of the HPP. Recent HPP questions involved issues around assessing MS quality, validating automated findings and considering potential alternative protein assignments for specific PSMs. Due to the evolution of HPP data submission guidelines and data re-analysis metrics, we have a higher baseline of proteins at PE2-4 levels from which ongoing discovery and transition to PE1 status continues. Current metrics for a protein to be PE1 are based on statistical calculations minimizing the risk that any peptide can be randomly mapped to multiple genes products.

Of the 20,055 currently allocated proteins in the human proteome (neXtProt 12 February 2016), only 16,518 were PE1, with a further 588 considered at best to be hypothetical (PE5). This means that at present 2,949 proteins are PE2-4; composed of 2,290 PE2 (transcript only), 565 PE3 (inferred from homology) and 94 PE4 (predicted). While only 2,949 PE2-4 proteins remain to be confirmed by high-stringency HPP metrics, our current approach takes little account of the potential goldmine of valid data available from other sectors of the scientific community. We argue that collectively alternative sources of complementary data provide clues that may facilitate the discovery of additional PE2-4 proteins by subsequent HPP MS metrics. Recognizing this fact, we acted upon comments made by researchers outside the proteomics community who argued that in order to be functional or biologically relevant a protein did not need to be reduced to any statistically required number of proteotypic peptides of any predefined length. As an example, they noted the many highly bioactive secretory peptides, such as neuropeptides, which were crucial to human biology. Several of these peptides are very short (<9 amino acids) secreted proteoforms that perform essential functions as intercellular signals. However, such peptides do not fall within the currently accepted thresholds in bottom-up HPP MS experiments. These constraints (that is, two uniquely mapping proteotypic peptides at least nine amino acids long) preclude discovery and annotation of these peptides as PE1, as well as incorporation into high-stringency datasets. Thus, short peptide proteoforms, such as the orexigenic neuropeptide QRFP, continue to be ‘missing' in HPP databases, annotated as known only at the transcript level ( https://www.nextprot.org/entry/NX_P83859/sequence). Similar arguments have been made about proteins unable to be cleaved by trypsin to produce two uniquely mapping proteotypic peptides of at least nine amino acids.

Analysis of Box 1 data reveals significant HPP progress. Over the period 2013–16, PE1 assignments have increased by 5% from 15,649 to 16,518 (78→82% of the estimated human proteome), with 1,079 PE2-5 entries re-assigned as PE1. This has occurred despite deliberate efforts to increase stringent MS metrics, leading to 432 PE1 proteins being downgraded to PE2-5 proteins. Interestingly, the data demonstrate that 22 new PE1 proteins were listed, which were previously not present at any PE level (for example, UMAD1, SULT1A4, MYH16).

Unfortunately, as can occur when ‘big data' is not endorsed through annual community jamboree/forums, experimental evidences and detailed rationales for such re-classifications are not currently made public nor are they easily accessible to non-experts. We therefore encourage establishing annual PE annotation/assignment jamborees, analogous to how the human genome project dealt with similar challenges.

**Figure 1:**
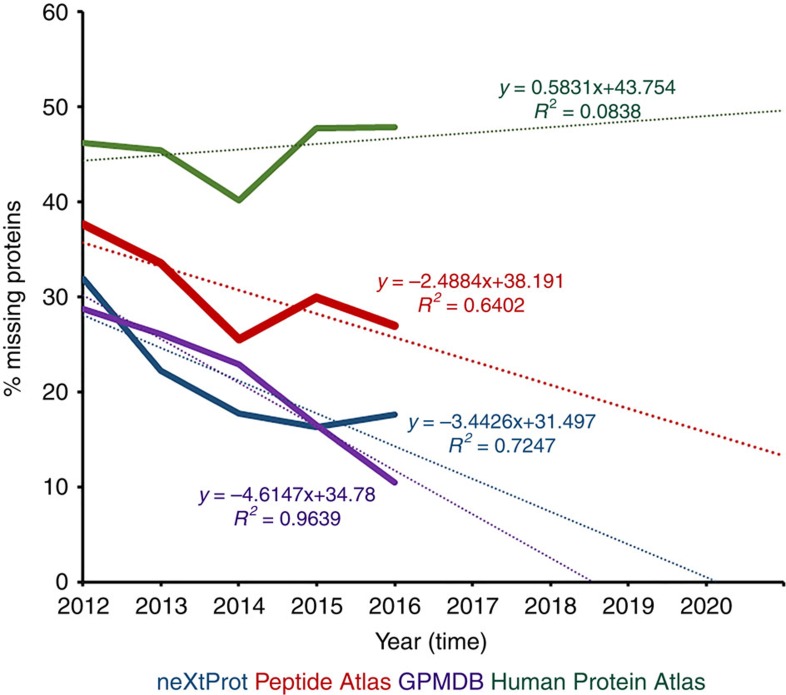
Extrapolation of linear best-fit rate equations demonstrates the rate at which various HPP input databases and GPMDB are currently ‘finding' PE2-4 proteins. Data required for this analysis (2012→2014) was extracted from Omenn *et al*.^4^ , with additional (2015 and 2016) statistics obtained from neXtProt, Peptide Atlas and GPMDB. Note: GPMDB data are not currently captured by neXtProt as part of the data input into the HPP (see Box 2), but GPMDB plays a role in defining annual HPP metrics.

Applying best fit linear extrapolations to all available PE re-assignment data^5^ (Fig. 1), it appears that with current neXtProt high-stringency metrics, the HPP will likely reach completion of ≥95% parts list coverage (PE1 status) near the close of the current decade (that is, 2020). As the final arbitrators of HPP PE1 calls, the statistical analysis of neXtProt is particularly telling, with a recent lag/hiatus evident. Equally, extrapolating PeptideAtlas data alone suggests 95% completion somewhere around 2030–40.

## Orthogonal efforts to find missing proteins

A major outcome from the C-HPP effort to date has been that researchers have been made to consider possible reasons why PE2-4 proteins have not been found by MS, Ab-based or other methods. This has now inspired the development of novel strategies to find the PE2-4 proteins, or understand why they are missing. Some approaches, envisaged to date, include subcellular enrichment of families, groups, clades or classes (for example, membrane proteins); more extensive protein and peptide fractionation before MS; increased MS accuracy, sensitivity and throughput; more reliable, specific and accurately validated Ab technologies, which are currently underway with collaborative efforts by the HPP Ab technology pillar; scrutiny of proteins not amenable to tryptic digestion, those failing to yield ‘flying' tryptic peptides or those outside observable mass range detection settings^14^; analysis of cross-linked or otherwise insoluble proteins; examination of rare human tissues/cells under differing spatiotemporal conditions or differentiation states; exposure of tissues to pathophysiological and/or environmental cues, and finally; broadening the capture of data from solely MS and Ab-based data streams.

## Bioinformatics efforts to understand missing proteins

**Figure 2:**
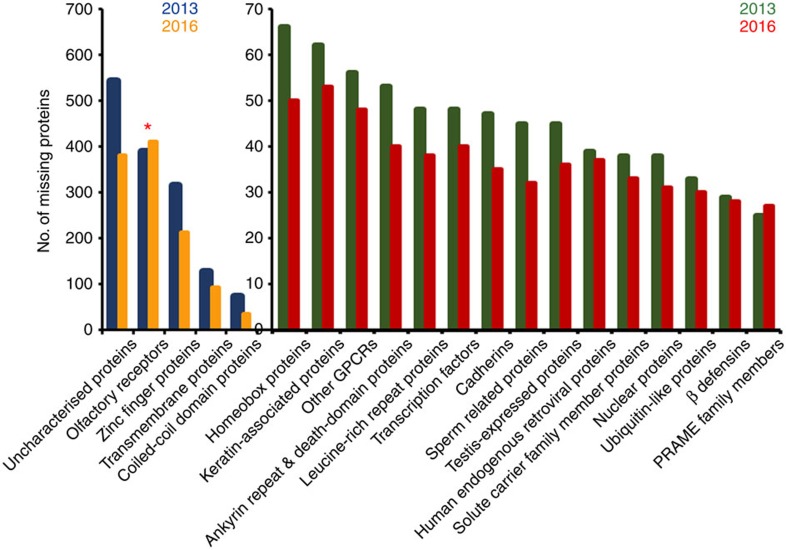
Top 20 missing protein families to determine protein families enriched in the February 2016 neXtProt PE2-4 report list. According to these data, olfactory receptors (ORs; marked with a red asterisk *) represent the largest family of PE2-4 proteins. The olfactory receptors also show the largest increase between 2013 and 2016 (that is, 15% in 2016 from 10% in 2013) when compared to the other families. The scale ‘0–70' represents a magnified axis scale for protein descriptors having <70 missing proteins. Blue and green colours represent PE2-4 proteins from 2013 whereas orange and red colours represent 2016 missing proteins.

**Figure 3:**
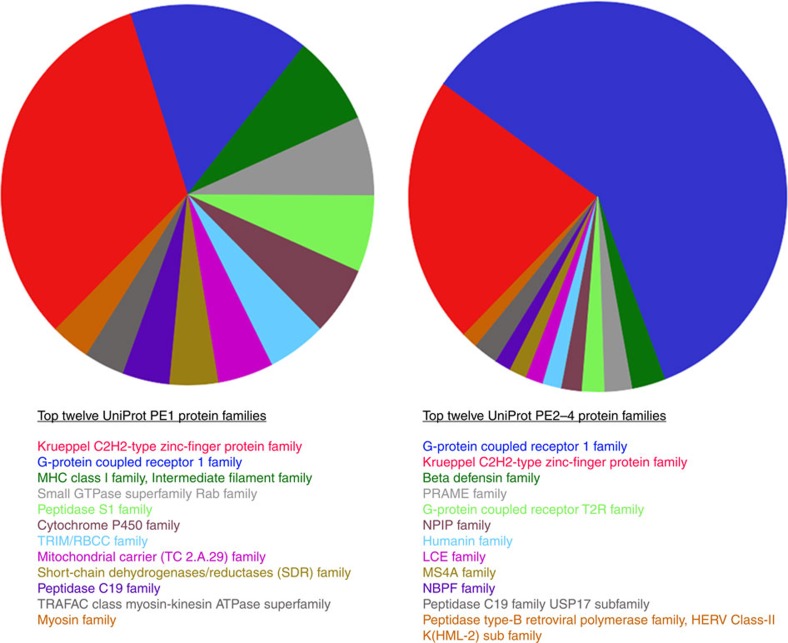
Most prolific PE1 and 12 PE2-4 UniProt protein families represented in the HPP neXtProt February 2016 release. The most represented PE1 families (left hand side) are the Krueppel zinc-finger protein family followed by the G-protein coupled receptor 1 family. These two families are also at the top of the PE2-4 category (right hand side) with the order reversed.

**Figure 4:**
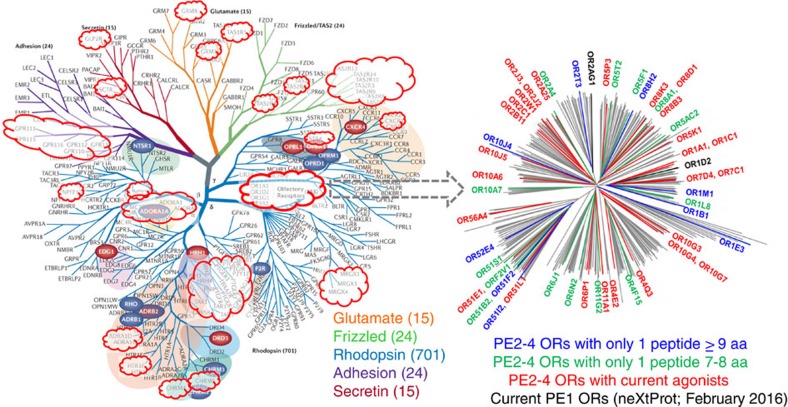
Phylogenetic analysis of PE distribution across GPCRs and olfactory receptors. In this composite figure, GPCR (left) family branches (largest ‘receptor' subset of all human and the PE2-4 proteins) are shown in an unrooted phylogenetic tree from Panther analyses with PE2-4 GPCRs highlighted inside red clouds, and an unrooted GCPR subset phylogenetic tree showing olfactory receptors (right) was produced using iTOP^56^, from neXtProt February 2016 PE1 olfactory receptors or best available, manually validated proteotypic MS evidence for olfactory receptor was retrieved. olfactory receptors with functional activity (known agonists) are shown in red in the left figure, as from Mainland *et al*.^16^. GPCR figure modified with permission from Macmillan Publishers Ltd: *Nature Reviews*. *Drug Discovery*, Stevens *et al*.^57^ copyright 2013.

Given the current scientific and protein informatics data detailed in Supplementary Table 1 and with a view to finding more PE2-4 proteins, we additionally undertook bioinformatics analyses of all PE2-4 proteins according to their families, sub-families, clades, groups, ontologies, pathways and networks. Figures 2, 3, 4 summarize these analyses with increasing depth across neXtProt descriptors (Fig. 2), comparison of protein biologies between PE1 and PE2-4 (Fig. 3), and PE2-4 G protein-coupled receptor (GPCR) family (Fig. 4, left) and OR* (Fig. 4, right) clade phylogenetic tree analyses, focussing on the most populous protein families from Figs 2 and 3.

Analyses of major descriptors (that is, protein subfamilies, classes, domain-type) for neXtProt 2016 PE2-4s indicated that five groups of proteins were highly represented. The PE2-4 groups with greater than 50 members in decreasing order are: olfactory receptors (red * in Fig. 2), zinc finger proteins, non-GPCR transmembrane proteins, coil-coil domain proteins and homeobox proteins (Fig. 2). Encouragingly, our analysis demonstrates a decrease in the percentage of HPP PE2-4 proteins assigned as ‘uncharacterized' by neXtProt over the 2013–16 period. These data also demonstrate the substantial success made across all major (that is, the top 20) protein groups, with the sole exception of the enigmatic olfactory receptors. In agreement with these data, Panther Protein Class analysis of 2,491 classifiable genes confirmed the major PE2-4 protein types were: receptors (PC00197), transcription factors (PC00218), transferases (PC00220), transporters (PC00227), membrane traffic proteins (PC00150), enzyme modulators (PC00095) and signalling molecules (PC00207), with other groups represented at low percentages.

Analysis of the top 12 UniProt families found in the 2016 PE2-4 and the PE1 lists (Fig. 3) demonstrates a highly significant enrichment of GPCR type 1 family missing proteins, and a reduction in the % of zinc finger proteins in the PE2-4 proteins list. Furthermore, we note that when the highest 12 families are examined in the PE2-4 list, the vast majority of those families' members are found to be ‘missing', with relatively few PE1 representatives. Only three families (that is, Kruppel C2H2-type zinc finger, GPCR type 1 and Peptidase C19 protein families) were common to both the major PE1 and the major PE2-4 families. Interestingly, PE1 assignments account for only 22% of all GPCR type 1 proteins while it accounts for 59% of the Kruppel zinc finger proteins. If one considers only the PE2-4 ‘missing' proteins, GPCR type 1 members represent 25% and zinc finger family members 9%. On a family-by-family basis, apart from Kruppel zinc finger (34%) and peptidase C19 (31%) proteins, the remainder of the top 12 families are noticeably composed of missing proteins (that is, range 50–95% of the total family membership). This implies that when a major family is ‘missing' by current HPP metrics, extremely limited high-stringency MS knowledge exists for any member of that protein family (for example, of 22 known PRAME proteins 19, 86% are assigned as PE2-4 and re-analysis of olfactory receptor MS data summarized in Supplementary Table 2 shows all (100%) are currently missing).

## The olfactory receptor family missing proteins

Subsequently, we examined the largest PE2-4 family, namely human GPCRs (shown in dark blue in Fig. 3). These are responsible for cellular responses to everything from protons and photons to hormones of >30 kd, metals, nutrients, small molecules including volatiles and neurotransmitters through many of our major senses (that is, sight, olfaction and taste). GPCRs also are the most important pharmaceutical drug target and largest family (>800) in the human proteome, as well as the largest membrane receptor family. They instigate signalling through nucleotide exchange involving heterotrimeric G-proteins and can be classified into five major families and subdivided into subfamilies based on sequence homology, to (1) rhodopsin (class A), (2) secretin, (3) adhesion (class B), (4) glutamate (class C), and (5) Frizzled/taste receptor 2 (TAS2). Phylogenetic analysis of GPCR PE2-4 proteins demonstrates that although singleton representatives and a few clusters are distributed across all five major subfamily branches/classes (Fig. 4), by far the highest proportion of missing proteins (*n*=400; ∼15% of all human PE2-4 proteins) emanate from the rhodopsin branch of the unrooted GPCR phylogenetic tree where the olfactory receptors reside. Note that family members with determined crystal structures are highlighted on the phylogenetic tree in coloured ovals (including ADORA2A, which has been recently re-classified by neXtProt as PE1).

Discovering functionality of the complete missing human olfactory receptor repertoire has proved difficult with only 49/∼400 human olfactory receptors having known ligands before the recent studies of Mainland *et al*.^16^. Using high-throughput screens of human olfactory receptors against 73 potential ligands they identified agonists for 27 receptors (coloured red in Fig. 4, right), including 18 that were previously orphan receptors. Their dataset addressed a bottleneck in research around functionality of human olfactory receptors by showing how physical olfaction stimuli can signal post-receptor activation. Correlating odorant ligands to olfactory receptors provides a valuable database, identifying functional olfactory receptors with potential to be strategically targeted through proteomic approaches and subsequent conversion to PE1 proteins.

The recent studies by Kim *et al,*^10^ and Wilhelm *et al*.^11^ generated intense interest in MS evidence for the expression of the chemosensory olfactory receptor family, as they claimed to have ‘unearthed' a surprisingly high number of 108 and 200 PE2-4 olfactory receptors, respectively. Of the human genome's 480 olfactory receptor genes in the latest version of neXtProt, 12 are considered hypothetical or putative (PE5). The remaining 468 olfactory receptor genes code for 411 unique proteins, with only two classified as PE1, and the remaining 409 classified as PE2-4. The claims for finding missing olfactory receptors by the draft human proteome papers above were rapidly critiqued by Ezkurdia *et al*.^12^ and Deutsch *et al,*^13^ on the basis of marginal spectral quality, deficiency of stringent protein/peptide 1% FDR criteria, use of short peptides, and erroneous or potentially ambiguous peptide identification, with the suggestion that these claims represent ‘the cream of false positives'. Collectively, these errors led Ezkurdia *et al*.^12^ and Deutsch *et al*.^13^ to conclude that there was little evidence for even a single olfactory receptor (including the two listed in previous releases of PeptideAtlas). Incidentally, 10 olfactory receptors were considered ‘found' by Choong *et al*.^17^ in the 2015 release of neXtProt with MS and Ab evidence. However, this evidence was considered insufficient for all these 10 olfactory receptors, suggesting that currently known olfactory receptor proteins may not possess sufficiently documented protein evidence in neXtProt.

From the amazing repertoire of 411 unique olfactory receptor proteins, only two are currently considered PE1 in the neXtProt 2016 release (namely, OR2AG1 and OR1D2; coloured black in Fig. 4, right). For OR1D2, no MS or Ab evidence is available, with three publications cited as functional evidence. For OR2AG1, neXtProt reports a single peptide 7 amino acids long, with no Ab evidence and functional evidence from two publications^18^,^19^. One of these studies^18^ equally reports function for another olfactory receptor, namely OR1F12 but this remains classified by neXtProt as PE4, whose status is based upon sequence homology. Thus, it appears that both these PE1 olfactory receptor proteins do not actually conform to HPP MS-based metrics and require closer community examination (Box 3), as does the way we consider functional/biological data as evidence for PE.

Olfactory receptors are involved under most physiological situations with odour recognition but have recently been shown to be expressed in multiple epithelial tissues with many potential chemosensory roles^20^,^21^,^22^. Criticisms of olfactory receptor restriction to nasal epithelial tissue are ill-advised^11^ and appear erroneous^12^,^20^,^21^,^22^. Given these data and the comprehensive olfactory receptor functional studies conducted by Mainland *et al*.^16^, we believe that a systematic capture of non-MS data and a communal re-assessment of all olfactory receptor PE assignments would be timely. To bring additional perspective to the olfactory receptor mêlée and to emphasize the challenges we face in finding the missing olfactory receptors by high-stringency MS, we undertook an analysis of all currently available raw olfactory receptor spectra from public repositories. This re-analysis reinforces that the best available MS data fail to provide high-stringency PE1 level proof for any GPCR olfactory receptor members using current metrics (Supplementary Fig. 1 and Supplementary Table 2). Despite 2,361 manuscripts revealed by an ‘olfactory receptor and human' PubMed keyword search, only piecemeal MS evidence for any human olfactory receptor is currently available.

To verify the *status quo*, we trawled public MS proteomic repositories (including GPMDB, PRIDE, ProteomicsDB, MAXQB and Human ProteinPedia), and aggregated 122,717 peptide MS entries (PSMs of length ≥7 aa), including many with multiple PE2-4 olfactory receptor observations. This collective dataset was processed through a semi-automated workflow (Supplementary Fig. 1), including manual spectral validation to filter reliable peptide assignments, with consideration of leucine/isoleucine ambiguity and BLAST analysis to account for possible single amino acid variations coding for peptides, as detailed elsewhere^23^. Briefly, the data (using Batch Peptide Match) identified 4,751 proteotypic olfactory receptor peptides (3.9%), following removal of non-proteotypic and decoy peptides. Of the proteotypic peptides, only 286 (6%) were tagged with a high search engine confidence value score by either SEQUEST, Mascot or MaxQuant. Finally, manual spectral validation (taking into consideration, noise, error rates (to matched peptide sequence), the run of B and Y singly charged ions, unassigned peaks and relative intensity of the spectrum) allowed us to sift out 64 high quality spectra for 24 peptides. As two overlapping peptides could be merged for a single olfactory receptor, this culminated in 23 unique olfactory receptor peptides. In summary, this analysis provided MS evidence for 23 of 409 missing olfactory receptors (5.6%).

The best available MS evidence for these 23 olfactory receptors is shown in Supplementary Table 2, and it includes peptides from GPMDB (1 green, 1 yellow and 5 red peptides), PRIDE (10 peptides) and ProteomicsDB (7 peptides). It should be noted that 14 PSMs represent a single 7–8 amino acid peptide, while 9 possess a single PSM of >9 amino acids. Proteins derived from matches were cross-referenced against HPA with no (zero) olfactory receptors found in the current (May 2016) high confidence HPA premium dataset. In addition, 13 peptides (Supplementary Table 2) were found to have complete or partial matches with 14 SRM peptides listed in the current version of SRMAtlas.

In summary, we demonstrate that many missing PE2-4 olfactory receptors possess single high-confidence PSM evidence, although best available MS spectra are insufficient to meet current HPP metrics. These could be considered as PE2-4 proteins ‘waiting in the wings', requiring confirmatory proteotypic PSM identifications at the required length to reach high-stringency requirements.

## Chromosome 7 example missing proteins

**Figure 5:**
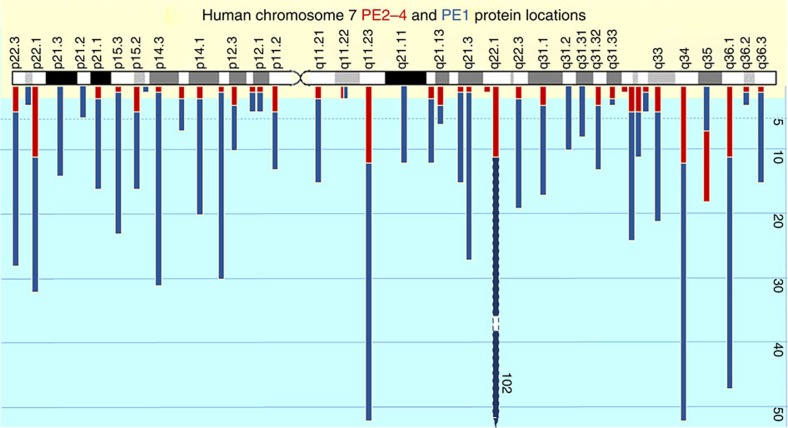
Positional mapping of the PE1 (757) and PE2-4 (139) proteins along human Chr 7. The data show random distribution of both along the complete length of human Chr 7. However, Giemsa banding patterns of light (GC-rich) and dark (GC-poor) bands are shown that debatably correspond to regions of gene density from light (higher gene density) to dark (lower gene density)^58^.

Under the C-HPP, the proteomic information found across chromosomes 1-22, X, Y and mitochondrial DNA are being studied by country-based or regional cluster teams. Australia and New Zealand undertook analysis of the proteins coded by human chromosome 7 (Chr 7)^24^,^25^. As part of our ongoing efforts, we demonstrate that current PE2-4 proteins are located across the length of the long and short arms, approximately equally dispersed across the length of Chr 7 (Fig. 5). This holds true for the majority (but not all) chromosomes examined to date. At one chromosomal location, namely 7q35, a significantly greater number of PE2-4 proteins (18/25) were found than PE1 proteins (7/25). Interestingly, however, when Giemsa (that is, reported relative gene richness) staining patterns along Chr 7 were compared for PE2-4 and PE1 distribution, we observed that 56% PE2-4s emanate from high gene density Chr 7 regions, 12% from moderate, 25% from low-moderate and only 1.5% from regions of low gene density. PE1 proteins generally distribute across Chr 7 locations with PE2-4 proteins, with few regions (only p22.2, p21.3, p21.2, p15.1, q21.11, q31.2 and q31.31) not having both PE classifications represented. Chr 7 PE2-4 proteins do not emanate from gene-poor regions and hence it is reasonable to suspect that other factors (for example, low spatiotemporal expression) are more likely to explain why they have not been found by high-stringency MS to date. These observations need to be replicated for all chromosomes by other C-HPP teams.

Of the 134 Chr 7 PE2-4 proteins, 27 are known to be GPCRs. The majority of these encode olfactory (15) or taste-related (six) receptors, with only four ‘orphan' GPCRs and two well-described GPCRs (5-HT_5A_ and mGluR8). There are many reasons why these proteins may still be considered missing. First, they all have restricted anatomical expression. In particular, the receptors for odours and ingested chemicals, which are likely expressed in only a few cells in specific regions of the body. Further, many missing proteins may be localized to a few discrete cells and/or difficult to access cellular compartments, like axon terminals, inner/outer hair cells (OHCs) or cilia on olfactory sensory neurones. Second, receptor expression may be extremely low even where they are physiologically active. Finally, it is possible that gene products are not translated/transcribed under normal physiological situations, or indeed at all. Their absence from proteomic databases suggests they are not highly abundant but it does not mean they are not important or not expressed. Indeed, a cursory examination of Chr 7 PE2-4 GPCR proteins reveals many non-proteomic studies show these GPCRs represent a very active part of the human proteome. Using the BPS/IUPHAR Concise Guide to Pharmacology ( http://www.guidetopharmacology.org/index.jsp)^26^ as a starting point for analysis, we provide some examples. First, HTR5A is part of the large family of receptors for the neurotransmitter serotonin (5-HT). When expressed, 5-HT_5A_ receptors stimulate G protein activity resulting in inhibition of adenylyl cyclase^27^, indicating it is a functional GPCR. mRNA for 5HT_5A_ receptor has been detected in the human brain by *in situ* hybridization^28^ and PCR^29^. However, our search shows no reports of protein localization by immunohistochemistry or identification by western blot in any human tissue. Mice with a 5-HT_5A_ receptor deletion have altered behaviour and a distinct response to the serotonin receptor ligand LSD^30^, indicating the protein is functional. It is likely that low levels of protein and restricted anatomical localization preclude identification of 5-HT_5A_ receptors by MS.

A second receptor we considered is GRM8 (metabotropic glutamate receptor 8, mGlu_8_), which is part of the large family of receptors for the prominent neurotransmitter glutamate. In a heterologous expression system, activation of mGlu_8_ receptors results in inhibition of adenylyl cyclase^31^, indicating it is a functional GPCR. *In situ* hybridization reveals discrete but low levels of mRNA in human brain^32^,^33^, while mGlu_8_ mRNA has been reported in cancer cell lines^34^, hippocampal cells^35^, astrocytes^36^ and in patient tissue in epilepsy or multiple sclerosis. Murine deletion of mGlu_8_ affects hippocampal synaptic transmission^37^, suggesting function under physiological conditions. Low levels and restricted anatomical localization may preclude identification of mGlu_8_ receptors by MS, although the receptor is also large and has a complex genetic structure, which probably leads to alternatively splice transcripts, and potentially several protein species^33^,^38^.

Finally, GPR22 (Probable G-protein coupled receptor 22) is a class A GPCR, with mRNA expressed in human heart and brain^39^,^40^,^41^,^42^. Interestingly, GPR22 has an unusually AT-rich mRNA, and only when enrichment is artificially rectified by introduction of G-C bases can signalling be restored in heterologous expression systems (Gi/o-mediated stimulation of G protein activity and constitutive inhibition of AC activity^41^). No ligand has been identified for GPR22, and GPR22 knockouts seem physiologically unremarkable. However, GPR22 mRNA is significantly reduced by aortic banding, a procedure that mimics cardiac hypertrophy produced by high blood pressure, and in GPR22 knockouts heart failure follows more rapidly than in wild type animals, implying a role for responses to cardiac stress^41^. There is no peer-reviewed report of GPR22 immunoreactivity in human tissues, although several corporate sites show neurons and other cells displaying putative GPR22 immunoreactivity. Sera from mice immunized against a human GPR22 peptide label cells in rat heart, although staining suggests GPR22 is restricted to subsets of myocytes^41^. The lack of an identified ligand for GPR22 has dampened enthusiasm for further pursuing functional studies through conventional biochemistry, and coupled with lack of neuronal phenotype in GPR22 null mice, it is not surprising no further attention has been paid to it. Unlike 5HT_5A_ and mGlu_8_ receptors, which likely have roles in normal physiology (even if understudied), there is little evidence to speak for or against function of GPR22, despite mRNA being detected by multiple investigators. However, for even the most obscure (non-olfactory) PE2-4 GPCRs, some evidence exists, suggesting that they are expressed in some tissues under certain conditions.

While we can learn much from an analysis of the Chr 7 PE2-4 GPCR proteins, the reasons for other proteins apparently ‘falling through the cracks' and having PE2-4 assignments may be legion. Below, we examine two current PE2-4 examples that appear to have strong biological non-HPP evidence that, combined with the olfactory receptor data above, argue for a broader, community-based, open data base strategy. We propose that opening up the HPP to consider other sources of data might concomitantly accelerate re-classification of PE2-4 proteins to PE1 status through the existing high-stringency HPP workflow.

In an orthogonal approach to understand the Chr 7 PE2-4 proteins, an example was randomly selected. Prestin (gene name *SLC26A5*) retrieved 91 peer-reviewed PubMed manuscripts, with the oldest in 2000 entitled ‘Prestin is the motor protein of cochlear outer hair cells'^43^, while another was a recent review of structural and functional properties^44^. Antibodypedia unearthed 83 anti-prestin Abs from 15 different vendors ( http://www.antibodypedia.com/explore/prestin). Though not listed on the Therapeutic Target database, Drugbank or Binding DB, prestin's substrates are listed as Cl^−^ and HCO^−^_3_ by the IUPHAR-DB (pharmacological targets) database^45^. Additionally, the Human Gene Mutation Database lists two prestin missense/nonsense mutations that produce deafness/autism phenotypes (CM075015 and CM124551), with one splice-variant linked with deafness (CS030995). Furthermore, the gene is known to have 15 transcripts. Equally, 12 patients with overlapping copy number variants are listed in DECIPHER: Database of Genomic variants and phenotype in Humans Using Ensembl Resources. Additionally, zebrafish studies captured in ZFIN include several CRISPR targeting agents ( http://zfin.org/ZDB-GENE-030131-1566) directed against prestin. In conclusion, this randomly selected Chr 7 PE2-4 protein shows there is copious public functional evidence at the protein level available, despite there being zero high-stringency MS or acceptable Ab evidence.

Particular physiological, cell and molecular factors make prestin intractable to being found by MS. First, it is a bullet-shaped membrane protein that is localized only on the OHCs of the mammalian inner ear^46^. This presents three challenges; highly specific tissue of origin, low copy number and membrane localization. OHCs are relatively few in number and are in the minority of the cells of the cochlea^47^, requiring specialized techniques such as laser capture microdissection to capture cells from very thin tissue sections. Each cochlear microdissection performed by Anderson *et al*.^47^ found only 200–300 OHCs per human being, far below the number required for routine proteomic analysis, let alone those involving OHC plasma membrane preparations. Equally, we know that membrane proteins are notoriously resistant to purification and identification by traditional techniques; requiring specialized enrichment strategies due to low copy number per cell, high-hydrophobicity and potential shielding of tryptic cleavage sites by either co-localized membrane proteins or the lipid bilayer itself. It is understandable why prestin is currently a PE2 (transcript evidence only) protein, even though 10 synthetic 10-28mer proteotypic peptides have been reported in neXtProt^45^, but no endogenous peptides have yet been captured experimentally by MS.

## Interleukin 9 an example missing protein

**Figure 6:**
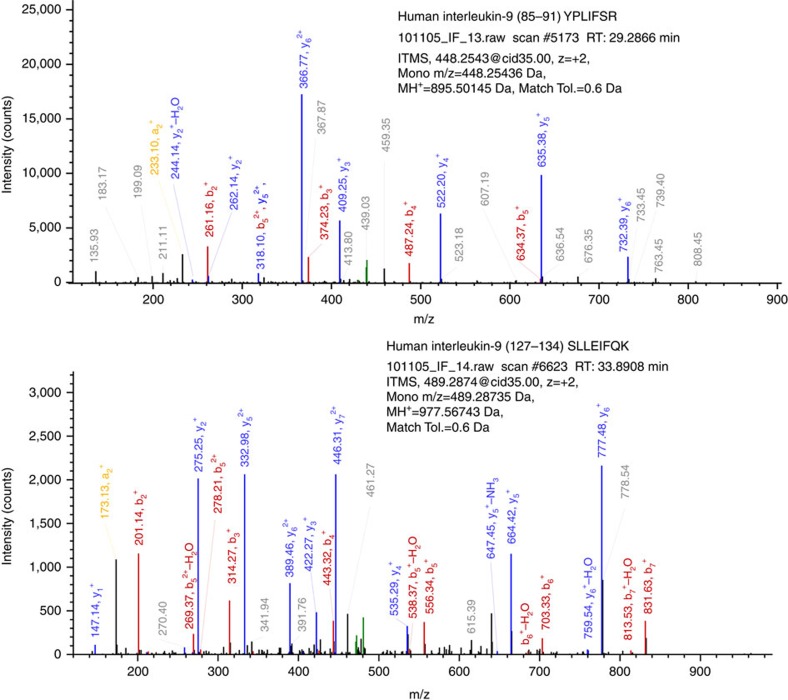
Fragmentation spectra of two IL-9 proteotypic peptides detected in the secretome of activated T-cells. Although not yet observed in any publicly available MS databases, both of these peptides are predicted to be proteotypic by neXtProt Unicity checker ( https://search.nextprot.org/viewers/unicity-checker/app/index.html).

A number of small biologically active secretory proteins risk being overlooked primarily because of their typical low abundance *in vivo* (in particular relative to the extremely high level of extracellular ‘background' proteins), in combination with a specific spatiotemporal expression/secretion profile, a very limited number of predicted potential proteotypic peptides and a relatively high ratio of post-translationally modified residues. One obvious example is the MS detection of interleukin-9 (IL-9) in secretome analysis of post-activation primary cultured T cells. Previous studies of the secretome of cells *ex vivo* had never identified IL-9, as they typically involve only short culture times. To facilitate secretome analysis, typical studies analyse cells grown in serum-free media, inevitably generating considerable cellular stress (with many stress- and apoptosis-related proteins detected). When we analysed cells grown for several days in the presence of foetal bovine serum (described in Supplementary Note 1), a very high percentage (≈95%) of detected tryptic peptides from the conditioned media proteins are evidently of bovine serum origin. After exclusion of bovine proteins and human T cell secretory proteins released from control ‘resting' (non-activated) cells, many other secretory proteins (for example, missing interleukins) are now exclusively detected from activated cells. Among these is the 125 amino acid residue, currently PE2 protein, IL-9. MS analyses reveal that IL-9 generates two proteotypic peptides of 7 and 8 residues, respectively (Fig. 6). Subsequent deposition of this and similar data into ProteomeXchange with annual communal re-analysis with stringent criteria will result in the re-classification of IL-9 as PE1. Similar discoveries accompanied with appropriate MS data deposition are expected to result in the re-classification of PE2-4 missing proteins that are unable to generate any proteotypic peptides acceptable to the HPP metrics, yielding a dramatic increase in the rates of discovery of missing proteins.

## Complementary efforts to characterize missing proteins

At present, there are also unrelated efforts (for example, Antibodypedia) to capture standardized, non-HPA affinity reagent data. Abs represent the main thrust one of the three pillars of the HPP initiative, and Ab-based techniques (for example Ab-enrichment, immunohistochemistry, western blot) support the search for the PE2-4 missing proteins^48^. However, issues around validity of Ab data have recently been raised across many forums, including this journal^49^. Key problems revolve around selectivity, acceptability and suitability for a given specific application. To facilitate resolving these issues, efforts are being made (for example, Antibodypedia, HPA) to collect, in searchable databases, detailed information concerning Ab validation and their use, and in some cases, literature performance review. Clearly, careful validation of all Abs is mandatory to allow researchers to make informed choices about suitable reagents with the knowledge that they are specific, selective, fit-for-purpose and reproducible in the context for which they are required^50^. Such validation should include western blot, immunohistochemistry, immunofluorescence, flow cytometry and microarrays, and ideally also Surface Plasmon Resonance data with detailed kinetic information. Where possible, the use of gene knockout/gene silencing (for example RNAi, CRISPR/Cas9) to confirm specificity has also been proposed^51^. Both polyclonal Abs (ideally affinity-purified) and monoclonal Abs have their advantages and disadvantages in the search for the PE2-4 missing proteins. Multiple epitopes, accessible by polyclonal Abs, can facilitate targeting specific proteins in complexes where some epitopes may be masked. They do, however, often have higher non-specific background and cannot be replaced once stocks are depleted. Monoclonal Abs, by contrast, are a renewable resource and typically have high affinity, high specificity and reduced non-specific binding^52^, while binding only a single epitope. Furthermore, monoclonal Ab libraries against target proteins can be readily generated^53^. For the missing proteins, a further dilemma is how to obtain an appropriate antigen for immunization. A potentially generic approach is the use of a proteospecific recombinant protein fragment and Protein Epitope Signature Tags (PrESTs)^54^. In a recent study, this approach has successfully generated a panel of monoclonal Abs and affinity purified polyclonal Abs against a number of targets, including some missing proteins^48^.

## MissingProteinPedia

The availability of large volumes of published, peer-reviewed, credible scientific data for PE2-4 proteins outside of high-stringency PE1 MS and Ab-based evidence (for example, IL-9 and prestin) struck us as a resource we could further exploit. Given the need to accelerate the HPP, we contend that the acquisition of such additional data streams concerning the biology of all PE2-4 proteins is self-evident. This has inspired us to explore, create and launch a communal database called MissingProteinPedia. This database assembles in one repository the vast amounts of publicly available, complementary data about all the current PE2-4 proteins that sit outside of the well-justified, high-stringency HPP pipeline. We contend that the knowledge captured by MissingProteinPedia will accelerate the communal HPP effort, as we seek strategies to allow the generation of high confidence MS evidence for as many PE2-4 proteins as possible. In addition, by providing an assembly of all available biological clues in one repository about every single current PE2-4 protein, it is likely that the MissingProteinPedia database may assist C-HPP chromosomal teams that have accepted the ‘Top 50 Missing Protein Marathon Challenge' launched recently at the 15th HUPO 2016 World Congress in Taipei to successfully identify an additional 50 PE2-4 proteins per chromosome to those already found by high stringency methods.

MissingProteinPedia is an open, comprehensive, communal, evidence-based, searchable and sortable (by chromosome, tissue and keywords) community knowledgebase, addressing the HPP's PE2-4 proteins. The launch of MissingProteinPedia aims to capture the broadest level of scientific data necessary to increase the rate at which PE2-4 proteins are validated. MissingProteinPedia represents a new community-based proteomics tool, analogous to human genome annotation jamborees^55^, where open big data contributions are invited from the broader scientific community regarding evidence for the existence of any missing protein. Unlike the high-stringency HPP data re-analysis, MissingProteinPedia makes no attempt to edit or judge the quality of submitted data, rather utilizing data to expose hidden possibilities not deposited into the current HUPO-accredited databases, including legacy lab books, unpublished works and data found in commercial/protected environments. It is anticipated that MissingProteinPedia collation will reveal clues that will contribute to an acceleration of high quality MS and qualified Ab data that allow confirmation beyond reasonable doubt of many of the current PE2-4 missing proteins. We believe MissingProteinPedia can cooperate and be easily integrated with high-stringency HPP data re-analysis, assisting the completion of the first phase of the HPP on schedule.

In summary, MissingProteinPedia aims to define, summarize and discuss all available data (including single proteotypic MS spectra) for the so-called missing proteins, emphasizing why they may be currently difficult to observe/find, using standard proteomics MS and Ab-based techniques.

## Conclusions and the way forward

The HPP was launched in 2010 and since then has grown organically with a general initial phase aimed at providing knowledge about the human proteome parts list. Progress has entailed the formation of a two-pronged strategy (C-HPP and B/D-HPP) culminating in the creation of guidelines and repositories (for example, ProteomeXchange) for MS and Ab-based (for example, HPA) data deposition; metrics for communal, annual MS re-analysis (for example, PeptideAtlas); categorization of the ∼20,000 basal components of the human proteome into PE levels (PE1-5; neXtProt); and forums for discussion and communication between research teams (for example, annual HUPO Congresses and HHP workshops).

The controversial release of the two draft human proteome papers^10^,^11^ has compelled researchers to recognize that the HPP is still in its infancy and much remains to be done. This is especially so with regard to the absence of a universally agreed long-term strategy for piloting the project into the future the capture of high-stringency data from all potential MS and Ab sources, capture of the breadth of other scientific human protein data to searchable knowledgebases, and finally the dissemination of the impact and success of the HPP to the public.

Of 20,055 human proteins (neXtProt, February 2016), 16,518 are PE1 (known), a further 2,949 are currently PE2-4 proteins (missing), while 588 PE5 proteins are considered only to be hypothetical. Current PE1-5 assignment strategies do not take into account all other alternative data streams available from the broader scientific community, preferentially relying on high-stringency MS data.

Analysis undertaken herein demonstrates that the rate of progress of the HPP in finding PE1 proteins needs to be accelerated in order to meet proposed HPP decadal plans. To hasten the progress of the current high-stringency HPP engine, we propose to capture other credible scientific data focussing on the PE2-4 missing proteins. This complementary engine is called the MissingProteinPedia and provides clues in the search for missing proteins, learning more about proteins that fall through the cracks of current data re-analysis. It is our hope that the communal MissingProteinPedia tool will allow researchers to better understand where, how, when and why PE2-4 proteins can be found. Capture of high-stringency data will populate the pool of PE1 proteins more readily and efficiently, building our knowledge of what it is to be human in strictly molecular terms.

## Data availability

The mass spectrometry proteomics data have been deposited to the ProteomeXchange Consortium via the PRIDE partner repository with the dataset identifier PXD005656.

## Additional information

**How to cite this article:** Baker, M. S. *et al*. Accelerating the search for the missing proteins in the human proteome. *Nat. Commun.* **8,** 14271 doi: 10.1038/ncomms14271 (2017).

**Publisher's note:** Springer Nature remains neutral with regard to jurisdictional claims in published maps and institutional affiliations.

## Supplementary Material

Supplementary Information Supplementary Figure, Supplementary Tables, Supplementary Note, and Supplementary References.

## neXtProt protein evidence (PE) definitions and 2013→2016 PE data comparison.

neXtProt assigns every one of the 20,055 human proteome proteins as either PE1-5, using evolving communal metrics that have become stricter to improve identification confidence. HPP PE status from 2013→2016 is shown below (with protein numbers indicated top left and right of boxes).

The PE2-4 proteins are now considered as the missing proteins since insufficient evidence has been produced as per the HPP metrics. The criteria for categorizing PE status, using data other than MS, remains to be communally defined. neXtProt protein data are constantly updated, so PE numbers vary with each version release.

## Integration of MissingProteinPedia with HPP.

The *MissingProteinPedia* is a publicly available protein data and information sharing web system that aims to collate any relevant data pertaining to any PE2-4 protein. At its core is a flexible schema-based database-driven web system allowing captures of all PE2-4 protein PubMed data, based upon gene and protein including synonyms. The database also allows unpublished, preliminary or proprietary data (for example, antibody, MS, cell biology and genetic studies) to be shared with collaborators via a protected interface.

Schema 1: The *MissingProteinPedia* collates and displays protein information from existing databases using various web services and application programming interfaces. Furthermore, the web interface allows researchers to collaborate and share data not available through other databases. The schema includes the recent illustration of the high-stringency HPP metrics engine^9^.

*MissingProteinPedia* facilitates HPP cross-disciplinary collaboration by providing a complementary, unfiltered, lower stringency perspective to both the HPP metrics and guidelines approaches, enabling community evaluation and scrutiny. *MissingProteinPedia* incorporates text mining technology to fetch and search accumulated UniProt, GeneCards, GeneRifs, PubMed and ProtAnnotator PE2-4 data. In addition, *MissingProteinPedia* summarize publicly available MS data from PRIDE, GPMDB, ProteomicsDB and MaxQB for relevant PE2-4 proteins. It also allows community users to annotate data and administrators to curate information before web publication.

## Accelerating discovery of the complete human proteome.

We recognize the tremendous achievement the Human Proteome Project has made since its 2010 launch by making available high-quality, communal MS (and other) data for ∼82% of the human proteome (February 2016).

To accelerate discovery of the 15% of the human proteome defined as the missing PE2-4 proteins, we recommend and encourage the following:

1. All proteomics practitioners, human researchers and human biology/medicine journals renew their efforts to observe current high-stringency HPP re-analysis *metrics* and researcher data submission *guidelines.*
2. All MS data should be incorporated into a single database (for example ProteomeXchange), including MS databases not currently captured, where data are provided transparently for any claim for a current PE2-4 protein.
3. The HPP should communally develop metrics and guidelines for processes by which they deal with all non-MS data sources. In particular, transparency around how protein evidence scoring for non-MS data needs to be communally accepted and reported.
4. An annual jamboree to evaluate and approve both MS and non-MS protein evidence reclassification proposals.
5. All possible biological data concerning the PE2-4 missing proteins to be comprehensively captured in *Missing-ProteinPedia*.
