# Supplementary material for: Accelerating the search for the missing proteins in the human proteome
Source: Nat Commun. 2017 Jan 24;8:14271. doi: 10.1038/ncomms14271 (PMC5286205; doi:10.1038/ncomms14271)
Supplement: Supplementary Information — Supplementary Figure, Supplementary Tables, Supplementary Note, and Supplementary References. [file ncomms14271-s1.pdf]

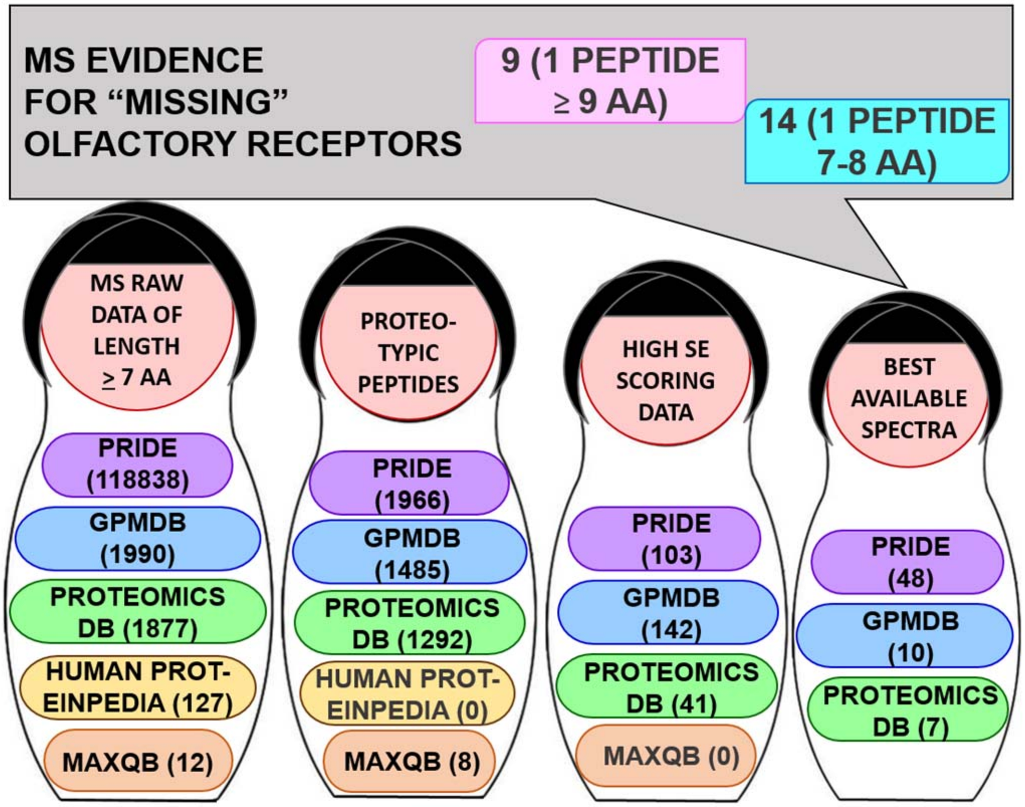

Supplementary Figure 1: Process used to hunt for missing human olfactory receptors using data from <u>many</u> publicly-available MS proteomic repositories, by computational identification of prototypic and high search engine (SE) scoring data for manual spectral quality check. Resulting best available spectral data were checked for sequence overlaps and then listed as most plausible of the best available MS evidence for missing ORs (details available in Supplementary Table 2).

Supplementary Table 1: Historical Efforts to Find the Missing Proteins – A PE2-4 HPP FactCheck (2012-current):

*Abbreviations: DAVID, **D**atabase for **A**nnotation, **V**isualisation and **I**ntegrated **D**iscovery; GRAVY; grand average of hydropathy; M_r, molecular mass; pI, isoelectric point; PRIDE, **Pr**oteomics **IDE**ntifications database; PXD; Proteome**X**change i**d**entifier.*

Missing Protein (PE2-4) Characteristic	Property or Question Addressed	Missing proteins/total human proteome (↑↓)	Ref.
Physicochemical Properties	pI, M_r, GRAVY, hydrophobicity	PE1/PE2 protein ratios: pI ↓ M_r ↑ GRAVY ↑ hydrophobicity ↓	1
	Hydrophobicity and transmembrane domains	1,224 were identified by modified 'hppK' analysis of human lymphoma cell lines out of which 2/3 were integral membrane proteins with 1-16 transmembrane segments. 13 missing proteins were identified which were previously in PE 2-5 categories. 7 out of these 13 proteins are Integral membrane proteins with 2-7 transmembrane domains. FDR 0.01 and validation based on q value	2
	Endopeptidase digestion	4 human proteins cannot produce a single proteotypic (tryptic) peptide with 36 not producing tryptic peptides in the 9-30 amino acid range usually "seen" by MS. No analysis using PE1 metrics (i.e., 2 x >9 amino acid proteotypic peptides) was conducted. Combination "Confetti" endopeptidase digestion predicted to increase coverage. Physical data comparisons difficult, as their definition (PE2-5) is not aligned with current HPP/neXtProt missing protein nomenclature (i.e., PE2-4 proteins are the missing proteins).	3
Protein Family, Subfamily or Biological Processes	Membrane (PXD✓)	Found 178 missing proteins expressed by 11 NSCL cancer cell lines, of which 74 were membrane proteins); 1% FDR ≥7aa, protein & peptide, GPCRs and Ig- like and P-loop containing NTPase zinc-finger proteins	4
	Membrane	PE5 proteins predicted (I-TASSER; COFACTOR) to be membrane/cell surface (transporter/receptor) and peptide fold families	5
	Tissue distribution, cellular components and biological processes	Manual curation of biological processes shows PE2-4 bias towards enriched zinc finger, GPCR (incl. olfactory receptors) and cadherin proteins. Data not condensed/quantitated. GO, Ingenuity IPA, neXtProt descriptor, PFAM and GPCR phylogeny remains to be done	6
Genetic or Evolutionary Aspects	Chromosomal Geography	http://proteomebrowser.org/tpb/home.jspx This web portal driven by the AANZ Chr 7 team, brings together data and information about human proteins from a number of sources and presents them in a gene- and chromosome-centric, interactive format	7
	Distribution PE2-4 across chromosomes	Genes for PE2-4 are not distributed evenly across human chromosomes or their regions. PE3/4 genes found outside conserved chromosomal regions. PE2-4 proteins tend to emerge at the telomere and centromere regions (more fragile experiencing breakage and rearrangement) and are adjacent to other protein coding genes but not adjacent to other PE-2-4 coding genes. PE2-4 gene "clusters" tend to have similar functional descriptors. PE3/4 genes have higher tendency to be "young" (related to latest common ancestor) and less conserved across different species	8

Missing Protein (PE2-4) Characteristic	Property or Question Addressed	Missing proteins/total human proteome (↑↓)	Ref.
Genetic or Evolutionary Aspects	Chromosomal	According to chromosomal files downloadable from neXtProt, PE2-4s are located on all 22 Chr pairs as well as X and Y, except MT. There are 2 MT proteins that fall into the PE1 category but lack proteomic (MS) evidence	9,10
	Chr12 PE2-4 chromosomal localisation	Suggested co-localisation of Chr12 PE2-4 proteins in close proximity to functionally-related genes, protein:protein interaction and disease networks. Patterns of PE2-4 gene localisation should be confirmed across other chromosomes	11
Organ, Tissue, Cell or Disease	Testis (PXD✓)	PE2-4s restricted to particular tissues (e.g., testis, post-meiotic germ cells). Found evidence for 89 PE2-4s, 1% peptide FDR; peptide length not mentioned, validated 3 PE5 proteins, twelve Chr2/14 PE2-4s found	12
	Testis (PXD✓)	3 human testis tissues; separated high and low Mr proteins; 166 PE2-4s identified by MS one ≥ 9aa and others ≥7aa, FDR,1% peptide & protein levels, transcriptomics shows PE2-4s; 108 PE2-4s (72% associated with disease (cancer)	13
	Testis	Testicular tissues - 2 Chr Y PE2-4s by Western blotting; no MS	14
	Retina/Placental	Examined 2 cell lines - one replicate from each, 58 of 74 multi-isoform genes are expressed at protein level	15
	Multi-tissues (PXD✓)	30 different tissues; 89 PE2-4s identified on Chr12; size of peptides not mentioned; 1% FDR peptide only	16
	HCC Cell lines	3 HCC cell lines with mRNA cell line analysis; small β-defensin (DEFB) PE2-4s' tryptic peptides will be very short; no other bias found; transcripts often not found as proteins	17
	Lymphoma Cell lines (PXD✓)	370 PE2-4s identified in any replicate, length of 1+ proteotypic peptide, length not mentioned; FDR <1% protein and PSM level, 32 PE2-4s identified across 3 replicates; only 4 PE2-4s when 2 proteotypic peptide across triplicates	18
	Lung adenocarcinoma (PXD✓)	Two PE2-4s identified; <1% peptide, ≥2 proteotypic peptides, length no mentioned	19
	CRC Tissues (PXD✓)	Claim 3,033 PE2-4s found <1% peptide FDR at 1 proteotypic peptide, protein FDR and peptide length not mentioned	20
	HCC cell lines	In 3 HCC cell lines, high % missing protein coding genes (especially Chr11) contain no mRNA identified evidence while Chr19 did show mRNA evidence. Need to shift search for to missing mRNAs	21
	Cancer cell lines (epigenetically modified) (iProx✓)	Found 19 PE2-4 proteins and 3 PE5 proteins, <1% peptide & protein FDR, a unique proteolytic peptide of ≥8aa. These proteins had no physicochemical differences with background proteins	22
	HCC cell line/healthy sera (PXD/iProx✓)	Separate high and low Mr subcellular fractionated proteins. 30 PE2-4 proteins identified at 1% FDR protein/peptide, >7aa from HCC cell lines (13) and from healthy sera (17), as well as 6 PE5 proteins	23

Missing Protein (PE2-4) Characteristic	Property or Question Addressed	Missing proteins/total human proteome (↑↓)	Ref.
Organ, Tissue, Cell or Disease	Breast cancer tissue (prior to HPP metrics)	Propose PE2-4 proteins likely due to very low-abundance and/or absence of expression in given cells or tissues. Proteome Discoverer and DAVID analyses show % PE1 and PE2-4 total and membrane proteins. Ingenuity pathways analyses (see update in current work) demonstrated PE2-4 proteins involved in molecular/cellular processes like lipid metabolism, small molecule biochemistry, cell-cell signalling, haematological function & immune cell trafficking. Propose subcellular membrane protein enrichment will improve low-abundance PE2-4 protein coverage	24
New Approach or Technology	Antibodypedia	Describe Antibodypedia as a curated, searchable database of Abs against human proteins. Ab coverage of the human genome graphically displayed on chromosome level (with validation and citation scoring criteria provided). 9% of human protein-coding genes lack Abs targeting any gene product and high redundancy in Abs against certain proteins exists. 5800 gene products have only 0–9 available Abs and are suggested as future focus	25
	Can PE2-4s be expressed and detected by MS	Cell-free in vitro transcription/translation IVTT allows expression of 18 missing proteins from Chr16	26
	MS CoPE2-4utational Analyses	Reanalyses from PRIDE identified 24 (17 were transmembrane proteins and 17 associated with secretory pathway) PE2-4s; 1+ proteotypic peptide, <1% protein FDR, peptide length not mentioned	27
	Multiple Spectral Library approach	Reanalysed placental PXD tissue dataset using multiple spectral libraries to identify 12 PE2-4 proteins, ≥two 7aa or one 9aa proteotypic peptides, FDRs not mentioned	28
	Modified MRM Approach (PXD✓)	Of 185 "targeted" PE2-4s 57 successfully detected using an MRM approach with synthetic peptide library	29
	Complete reanalysis of neXtProt	145 human proteins not expected to have proteotypic tryptic peptides (77 PE2-4, 58 PE1 and 10 PE5 proteins); length of peptides not mentioned. 58 neXtProt PE1 proteins that do not produce tryptic peptides emanate from highly similar protein evidence (40), experimental/functional characterisation (11), 3D structure (6) and binary interaction (1). Authors suggest need for review and possible use of Confetti, Ab and top-down MS approaches for proteins where tryptic digestion not anticipated	30
	CoPE2-4lete reanalysis of Chr12 neXtProt, GPMDB, PeptideAtlas, HPA & Ensembl data	1,066 protein coding genes identified, including 171 PE2-4s (only 2 by MS, no metrics provided)	31
	Functional annotation of neXtProt PE2-4 Chr7	New protocol integrating bioinformatics analysis and annotation tools, reporting mammalian homologues from sequential BLAST searches for 128 Chr 7 PE2-4 proteins and functional motifs, gene ontology and/or pathway analysis for another 27 proteins	32

Missing Protein (PE2-4) Characteristic	Property or Question Addressed	Missing proteins/total human proteome (↑↓)	Ref.
	Reanalysis of neXtProt PE2-4 Chr2 & 14 (PXD✓)	Informatics reanalysis of neXtProt data found 58 Chr2 & 14 PE2-4 proteins using >6aa, MASCOT ion score ≥30, 1% peptide FDR, SRM with synthetic peptides confirmation. Suggest PE1 status be confirmed.	33
	ProtAnnotator: functional annotation of all neXtProt PE2-4	www.biolinfo.org/protannotator Semi-automated pipeline, extending the bioinformatics approach in ref. 32. Homologues identified for 66%of PE2-4 proteins, with functional annotation for 51%.	34

Supplementary Table 2: Currently assigned PE2-4 missing ORs with manually-curated, best-available proteotypic peptide MS spectra.

Details of neXtProt identifier (ID), chromosome (Chr) number, peptide identified, with its length and start and end positions on protein sequence, number of MS observations (Obs.) and database(s) where peptide spectral information has been deposited.

#	neXtProt ID	Chr	Gene Name	Peptides identified	Peptide length	Peptide position	No of Obs	Database/s Used
ORs that **currently qualify** as PE1 proteins according to neXtProt Feb 2016 HPP high-stringency metrics (≥ 2 uniquely mapping peptides ≥ 9 residues)								
NIL								
ORs that **previously qualified** as PE1 proteins according to 2015 HPP neXtProt metrics (≥ 2 uniquely mapping peptides ≥ 7 residues or 1 uniquely mapping peptide ≥ 9 residues)								
1	NX_Q8NGR6	9	OR1B1	**IGAAILRLPSAAGR**[@]	14	227-240	1	GPMDB (yellow)
2	NX_Q8NGA1	19	OR1M1	**ILVAIMK**VPSAGGR[*,#]	14	221-234	2	GPMDB (both green)
3	NX_Q8NGH9	11	OR52E4	**TISILASVVVGR**[#]	12	142-153	1	PRIDE
4	NX_Q9H344	11	OR51I2	SVMATASREER	11	224-236	4	PRIDE
5	NX_Q8NH03	1	OR2T3	***SAAGHR***KAL[#]	9	235-243	2	GPMDB (red)
6	NX_P0C629	1	OR10J4	DALLRALGR	9	299-307	1	PRIDE
7	NX_Q8NH61	11	OR51F2	LYVVAVSGN	9	48-56	1	GPMDB (red)
8	NX_Q8N162	11	OR8H2	NAVIRVMQR	9	299-308	1	PRIDE
9	NX_Q8WZA6	17	OR1E3	**VPSTGGIQK**[#]	9	228-236	2	PRIDE
ORs that **did not previously and do not currently qualify** as PE1 proteins according to 2016 HPP neXtProt high-stringency metrics (i.e., only 1 uniquely mapping 7-8 residue peptide)								
10	NX_O95047	6	OR2A4	***GDNITSIR***[#]	8	2-9	1	GPMDB (red)
11	NX_Q9Y5P1	11	OR51B2	**QIQYGIIR**[#]	8	296-303	1	GPMDB (red)
12	NX_O95221	11	OR5F1	**ALANVISR**[#]	8	300-307	1	PROTEOMICSDB
13	NX_Q8NGG7	11	OR8A1	EVKAAVQK	8	312-319	1	PROTEOMICSDB
14	NX_Q8NGG2	11	OR5T2	**FVLDFNMK**[#]	8	36-43	1	GPMDB (red)
15	NX_Q8NGJ8	11	OR51S1	ILNRLQPR	8	310-317	1	PRIDE
16	NX_Q9H2C8	11	OR51V1	**IGLTIIGR**[#]	8	153-160	1	PROTEOMICSDB
17	NX_Q8NGE5	12	OR10A7	TVTLLGNF	8	36-43	1	PROTEOMICSDB
18	NX_Q8NGC5	14	OR6J1	MRAVLRSR	8	315-322	1	PRIDE
19	NX_Q8NGB8	15	OR4F15	KHKAISFR	8	88-95	2	PRIDE
20	NX_Q8NGY6	1	OR6N2	**IIGAVLK**[#]	7	221-227	1	PROTEOMICSDB
21	NX_Q9NZP5	3	OR5AC2	**VLFDILK**[#]	7	223-229	35	PRIDE, PROTEOMICSDB
22	NX_Q8NGR8	9	OR1L8	**ILTTVLK**[#]	7	222-228	1	PROTEOMICSDB
23	NX_Q8NGC1	14	OR11G2	DMRKALK	7	334-340	1	PRIDE

* overlapping peptides with overlap underlined

[#] matches with SRM peptides shown in bold font, with partial overlaps shown in italics

[@] contains two complete SRM peptides

Supplementary Note 1: Brief description of the IL-9 proteomics experiment

We carried out a secretome analysis of activated human T cells by performing a label-free bottom-up proteomic study of the conditioned medium of a primary cell culture. Viable naïve T cells were isolated as described before[35]. In order to minimize cell stress and to maintain them in good condition for several days, cells were cultured in conventional DMEM/F12 culture medium supplemented with 7.5% fetal bovine serum (FBS). Reduced and iodoacetamide alkylated proteins from the harvested cell supernatant were prefractionated on a C5 column, digested with trypsin and further analysed by data dependent nanoLC tandem MS on an ion trap/OrbiTrap mass spectrometer (LTQ OrbiTrap Velos (Thermo Fisher Scientific)). Conditioned media of activated as well as of resting T cells were collected at 6, 24 and 48 hours after the time of activation, and analysed in duplicate. The experiment yielded approximately 340,000 peptide spectral matches (representing 20% of all 1.69 million tandem MS spectra recorded). Nearly 320,000 of these PSMs could be mapped to just over 100 bovine (i.e. FBS derived) proteins. Slightly less than 21,000 PSMs uniquely mapped to a little over 500 human protein groups. Only in the medium of the 48h activated T cells, PSMs referring to human IL-9 were detected, yielding the representative MS/MS spectra shown in Figure 6.

Supplementary References:

1 Xu, S. *et al.* Appraisal of the Missing Proteins Based on the mRNAs Bound to Ribosomes. *Journal of Proteome Research* **14**, 4976-4984, doi:10.1021/acs.jproteome.5b00476 (2015).

2 Vit, O. *et al.* Large-scale identification of membrane proteins based on analysis of trypsin-protected transmembrane segments. *Journal of Proteomics*, doi:10.1016/j.jprot.2016.03.016 (2016).

3 Elguoshy, A. *et al.* Why are they missing? : Bioinformatics characterization of missing human proteins. *Journal of Proteomics*, doi:10.1016/j.jprot.2016.08.005 (2016).

4 Kitata, R. B. *et al.* Mining Missing Membrane Proteins by High-pH Reverse-Phase StageTip Fractionation and Multiple Reaction Monitoring Mass Spectrometry. *Journal of Proteome Research* **14**, 3658-3669, doi:10.1021/acs.jproteome.5b00477 (2015).

5 Dong, Q., Menon, R., Omenn, G. S. & Zhang, Y. Structural Bioinformatics Inspection of neXtProt PE5 Proteins in the Human Proteome. *Journal of Proteome Research* **14**, 3750-3761, doi:10.1021/acs.jproteome.5b00516 (2015).

6 Guruceaga, E., Sanchez del Pino, M. M., Corrales, F. J. & Segura, V. Prediction of a missing protein expression map in the context of the human proteome project. *Journal of Proteome Research* **14**, 1350-1360, doi:10.1021/pr500850u (2015).

7 Goode, R. J. *et al.* The proteome browser web portal. *Journal of Proteome Research* **12**, 172-178, doi:10.1021/pr3010056 (2013).

8 Xu, A. *et al.* Evolutionary Characteristics of Missing Proteins: Insights into the Evolution of Human Chromosomes Related to Missing-Protein-Encoding Genes. *Journal of Proteome Research* **14**, 4985-4994, doi:10.1021/acs.jproteome.5b00450 (2015).

9 Gaudet, P. *et al.* neXtProt: organizing protein knowledge in the context of human proteome projects. *Journal of Proteome Research* **12**, 293-298, doi:10.1021/pr300830v (2013).

10 Lane, L. *et al.* neXtProt: a knowledge platform for human proteins. *Nucleic acids research* **40**, D76-83, doi:10.1093/nar/gkr1179 (2012).

11 Jayaram, S. *et al.* Insights from Chromosome-Centric Mapping of Disease-Associated Genes: Chromosome 12 Perspective. *Journal of Proteome Research* **14**, 3432-3440, doi:10.1021/acs.jproteome.5b00488 (2015).

12 Jumeau, F. *et al.* Human Spermatozoa as a Model for Detecting Missing Proteins in the Context of the Chromosome-Centric Human Proteome Project. *Journal of Proteome Research* **14**, 3606-3620, doi:10.1021/acs.jproteome.5b00170 (2015).

13 Zhang, Y. *et al.* Tissue-Based Proteogenomics Reveals that Human Testis Endows Plentiful Missing Proteins. *Journal of Proteome Research* **14**, 3583-3594, doi:10.1021/acs.jproteome.5b00435 (2015).

14 Ahmadi Rastegar, D. *et al.* Isoform-Level Gene Expression Profiles of Human Y Chromosome Azoospermia Factor Genes and Their X Chromosome Paralogs in the Testicular Tissue of Non-Obstructive Azoospermia Patients. *Journal of Proteome Research* **14**, 3595-3605, doi:10.1021/acs.jproteome.5b00520 (2015).

15 Li, H. D. *et al.* Functional Networks of Highest-Connected Splice Isoforms: From The Chromosome 17 Human Proteome Project. *Journal of Proteome Research* **14**, 3484-3491, doi:10.1021/acs.jproteome.5b00494 (2015).

16 Manda, S. S. *et al.* Identification and characterization of proteins encoded by chromosome 12 as part of chromosome-centric human proteome project. *Journal of Proteome Research* **13**, 3166-3177, doi:10.1021/pr401123v (2014).

17 Fan, Y. *et al.* Insights from ENCODE on Missing Proteins: Why beta-Defensin Expression Is Scarcely Detected. *Journal of Proteome Research* **14**, 3635-3644, doi:10.1021/acs.jproteome.5b00565 (2015).

18 Diez, P. *et al.* Integration of Proteomics and Transcriptomics Data Sets for the Analysis of a Lymphoma B-Cell Line in the Context of the Chromosome-Centric Human Proteome Project. *Journal of Proteome Research* **14**, 3530-3540, doi:10.1021/acs.jproteome.5b00474 (2015).

19 Kim, Y. I. *et al.* Proteogenomic Study beyond Chromosome 9: New Insight into Expressed Variant Proteome and Transcriptome in Human Lung Adenocarcinoma Tissues. *Journal of Proteome Research* **14**, 5007-5016, doi:10.1021/acs.jproteome.5b00544 (2015).

20 Shiromizu, T. *et al.* Identification of missing proteins in the neXtProt database and unregistered phosphopeptides in the PhosphoSitePlus database as part of the Chromosome-centric Human Proteome Project. *Journal of Proteome Research* **12**, 2414-2421, doi:10.1021/pr300825v (2013).

21 Kong, N. *et al.* Assessing Transcription Regulatory Elements To Evaluate the Expression Status of Missing Protein Genes on Chromosomes 11 and 19. *Journal of Proteome Research* **14**, 4967-4975, doi:10.1021/acs.jproteome.5b00567 (2015).

22 Yang, L. *et al.* Finding Missing Proteins from the Epigenetically Manipulated Human Cell with Stringent Quality Criteria. *Journal of Proteome Research* **14**, 3645-3657, doi:10.1021/acs.jproteome.5b00480 (2015).

23 Su, N. *et al.* Special Enrichment Strategies Greatly Increase the Efficiency of Missing Proteins Identification from Regular Proteome Samples. *Journal of Proteome Research* **14**, 3680-3692, doi:10.1021/acs.jproteome.5b00481 (2015).

24 Muraoka, S. *et al.* In-depth membrane proteomic study of breast cancer tissues for the generation of a chromosome-based protein list. *Journal of Proteome Research* **12**, 208-213, doi:10.1021/pr300824m (2013).

25 Alm, T., von Feilitzen, K., Lundberg, E., Sivertsson, A. & Uhlen, M. A chromosome-centric analysis of antibodies directed toward the human proteome using Antibodypedia. *Journal of Proteome Research* **13**, 1669-1676, doi:10.1021/pr4011525 (2014).

26 Horvatovich, P. *et al.* In Vitro Transcription/Translation System: A Versatile Tool in the Search for Missing Proteins. *Journal of Proteome Research* **14**, 3441-3451, doi:10.1021/acs.jproteome.5b00486 (2015).

27 Kumar, D., Jain, A. & Dash, D. Probing the Missing Human Proteome: A Computational Perspective. *Journal of Proteome Research* **14**, 4949-4958, doi:10.1021/acs.jproteome.5b00728 (2015).

28 Cho, J. Y. *et al.* Combination of Multiple Spectral Libraries Improves the Current Search Methods Used to Identify Missing Proteins in the Chromosome-Centric Human Proteome Project. *Journal of Proteome Research* **14**, 4959-4966, doi:10.1021/acs.jproteome.5b00578 (2015).

29 Chen, C. *et al.* Screening of missing proteins in the human liver proteome by improved MRM-approach-based targeted proteomics. *Journal of Proteome Research* **13**, 1969-1978, doi:10.1021/pr4010986 (2014).

30 Choong, W. K. *et al.* Informatics View on the Challenges of Identifying Missing Proteins from Shotgun Proteomics. *Journal of Proteome Research* **14**, 5396-5407, doi:10.1021/acs.jproteome.5b00482 (2015).

31 Gupta, M. K. *et al.* Chromosome-centric human proteome project: deciphering proteins associated with glioma and neurodegenerative disorders on chromosome 12. *Journal of Proteome Research* **13**, 3178-3190, doi:10.1021/pr500023p (2014).

32 Ranganathan, S. *et al.* Functional annotation of the human chromosome 7 "missing"proteins: a bioinformatics approach. *Journal of Proteome Research* **12,** 2504-2510, doi: 10.1021/pr301082p.

33 Carapito, C. *et al.* Computational and Mass-Spectrometry-Based Workflow for the Discovery and Validation of Missing Human Proteins: Application to Chromosomes 2 and 14. *Journal of Proteome Research* **14**, 3621-3634, doi:10.1021/pr5010345 (2015).

34 Islam, M. T. *et al.* Protannotator: a semiautomated pipeline for chromosome-wise functional annotation of the "missing" human proteome. *Journal of Proteome Research* **13,** 76-83, doi: 10.1021/pr400794x.

35 Finoulst, I. *et al.* Identification of low abundant secreted proteins and peptides from primary culture supernatants of human T-cells. *Journal of Proteomics* **75**, 23-33, doi: 10.1016/j.jprot.2011.03.034 (2011).
